# Multiple cross displacement amplification coupled with a lateral flow biosensor for ultra-rapid and highly sensitive detection of *Escherichia coli* in neonatal sepsis

**DOI:** 10.1128/spectrum.04180-25

**Published:** 2026-06-15

**Authors:** Ruoyi Xiao, Peicen Zou, Juan Zhou, Ying Li, Ying Chen, Pan Huang, Yi Wang, Yajuan Wang

**Affiliations:** 1 Capital Institute of Pediatrics, Chinese Academy of Medical Sciences & Peking Union Medical College 36776, Beijing, China; 2 Department of Neonatology, Capital Center for Children’s Health, Capital Medical University 12517, Beijing, China; 3 Experimental Research Center, Capital Center for Children’s Health, Capital Medical University 12517, Beijing, China; University of Georgia College of Veterinary Medicine, Athens, Georgia, USA

**Keywords:** *Escherichia coli*, neonatal sepsis, multiple cross displacement amplification, lateral flow biosensor, rapid detection

## Abstract

*Escherichia coli* (*E. coli*) is a predominant pathogen causing neonatal sepsis, and rapid pathogen identification is critical for timely antimicrobial therapy. However, conventional diagnostic methods are often limited by prolonged turnaround time, or insufficient sensitivity. In this study, we developed a multiple cross-displacement amplification assay coupled with a lateral flow biosensor (MCDA-LFB) targeting the *uidA* gene for rapid visual detection of *E. coli*. Using a set of 10 specific primers, the reaction conditions were optimized at 63°C for 40 min. The analytical limit of detection was 50 fg genomic DNA per reaction, and no cross-reactivity was observed among the tested non–*E*. *coli* strains. Clinical performance was evaluated using 75 residual sterile body fluid specimens, including 58 blood and 17 cerebrospinal fluid samples, collected from neonates who were clinically diagnosed with sepsis or bacterial meningitis, and in whom *E. coli* infection was considered highly likely on the basis of the overall clinical assessment. Results were compared with quantitative PCR (qPCR) and conventional culture. In this cohort, the MCDA-LFB assay showed complete agreement with qPCR (*κ* = 1.0) and detected 56.0% (42/75) positive samples, compared with 14.7% (11/75) by culture (*P* < 0.001). The entire workflow, including nucleic acid extraction, amplification, and visual readout, was completed within approximately 1 h. These findings suggest that the *E. coli*-MCDA-LFB assay provides a rapid, sensitive, and cost-effective approach for *E. coli* detection and may be useful for early diagnosis in clinical neonatal sepsis, particularly in point-of-care and resource-limited settings.

**IMPORTANCE:**

Neonatal sepsis requires rapid and accurate pathogen identification to support timely antimicrobial therapy, yet conventional blood culture is often slow and has limited sensitivity, particularly in neonates with small sample volume or prior antibiotic exposure. In this study, we developed a visual multiple cross displacement amplification–lateral flow biosensor (MCDA-LFB) assay for rapid detection of *Escherichia coli*, a leading cause of neonatal sepsis. The assay enables visual readout within approximately 1 h, shows femtogram-level analytical sensitivity, and demonstrates complete agreement with quantitative PCR (qPCR) in our clinical validation cohort. Owing to its operational simplicity and minimal equipment requirements, this method may serve as a useful approach for rapid detection of neonatal *E. coli* infection, particularly in resource-limited clinical microbiology settings.

## INTRODUCTION

*Escherichia coli (E. coli*) is the predominant gram-negative bacterium responsible for neonatal infections globally. It is recognized as a major cause of severe neonatal infectious diseases, including sepsis and meningitis, particularly in preterm and low-birth-weight infants, where it drives high morbidity and mortality rates (1, 2). The estimated global incidence of neonatal *E. coli* infection ranges from 0.2 to 5 per 1,000 live births (3). Data from the U.S. NICHD Neonatal Research Network (2020) identify *E. coli* (36.6%) and Group B *Streptococcus* (30.2%) as the leading causes of early-onset sepsis, with *E. coli* infections affecting primarily preterm infants (4). Recent studies highlight an increasing incidence and rising antimicrobial resistance in neonatal *E. coli* infections, posing a growing threat to neonatal health (4–9). Early clinical manifestations, including feeding intolerance, lethargy, temperature instability, and tachypnea, are typically nonspecific (10). In severe cases, the illness may rapidly progress to septic shock or meningitis characterized by seizures (11). Consequently, neonatal *E. coli* infection is often clinically indistinguishable from other causes of infections, making timely etiologic diagnosis challenging. Therefore, early, rapid, and accurate pathogen identification is essential for guiding targeted antimicrobial therapy, reducing complications, and improving outcomes in affected neonates (12).

Current laboratory diagnostic approaches include conventional culture, matrix-assisted laser desorption/ionization time-of-flight mass spectrometry (MALDI-TOF MS), quantitative PCR (qPCR), digital PCR (dPCR), and metagenomic next-generation sequencing (mNGS). Blood culture remains the diagnostic standard, yet its utility is limited by prolonged turnaround times and suboptimal sensitivity. In neonates, restricted blood volumes, prior antibiotic exposure, and intermittent bacteremia contribute to low positivity rates and delayed reporting (13). While MALDI-TOF MS enables accurate identification, its dependence on culture growth constitutes a fundamental limitation (14, 15). Molecular methods offer advantages in speed and specificity^16^, ^17^; for instance, dPCR outperforms culture in low-copy-number samples (18). However, these platforms require specialized infrastructure, technical expertise, substantial cost, and lack standardized interpretation thresholds (19). Emerging sequencing techniques like mNGS provide complementary value in complex infections but are restricted by cost, background contamination, and data interpretation challenges (20). These limitations underscore the urgent clinical need for a rapid, sensitive, and operationally simple assay for targeted pathogen detection in neonatal infection.

Multiple cross displacement amplification (MCDA), a novel isothermal amplification technology, has been widely applied for pathogen detection due to its high sensitivity, speed, and simplicity (21–30). Utilizing strand-displacement polymerization and target-specific primers, MCDA enables efficient nucleic acid amplification under isothermal conditions, achieving femtogram-level sensitivity (31). Unlike PCR, MCDA relies only on a simple heating device. Furthermore, the method supports various readout strategies, including real-time turbidimetry and lateral flow biosensors (LFBs), providing direct visual detection. These formats allow immediate result visualization without agarose gel electrophoresis or sequencing, substantially shortening the time required for pathogen identification (23, 32).

While MCDA-LFB has been applied to other pathogens, no clinically validated protocol specifically targets *E. coli* in neonatal sepsis. The *uidA* gene, encoding β-glucuronidase, is highly conserved among *Escherichia coli* strains and has been validated in multiple molecular studies as a reliable molecular target for PCR amplification. Original work by Feng et al. demonstrated that *uidA* sequences are detectable in the vast majority of *E. coli* isolates, supporting its utility as a molecular marker (33). Owing to its robust conservation and specificity, *uidA* continues to be widely employed in diagnostic PCR and related nucleic acid amplification assays for the identification of *E. coli* in clinical diagnostic settings (34, 35). Consequently, this study established a simple, rapid, and visually interpretable *E. coli*-MCDA-LFB assay targeting *uidA*. We aimed to evaluate the analytical feasibility, sensitivity, and specificity of this assay, alongside its clinical performance. Additionally, we assessed its potential for early pathogen identification and its applicability in resource-limited settings, offering a promising tool to improve the diagnosis of neonatal *E. coli* infection.

## MATERIALS AND METHODS

### Reagents and instruments

Primers and labeled primers were synthesized by Tianyihuiyuan Biotech Co., Ltd (Beijing, China). The DNA isothermal amplification kit, lateral flow nanobiosensor (LFB), and visual detection reagent (VDR) were all supplied by Huidexin Biotech Co., Ltd (Tianjin, China). The nucleic acid extraction and purification kit was purchased from Novazene Biotech Co., Ltd (Nanjing, China). The real-time turbidimeter (LA-320C) was purchased from Eiken Chemical Co., Ltd (Tokyo, Japan). The NanoDrop 1000 micro-volume spectrophotometer was provided by Thermo Fisher Scientific Inc (USA), and the ABI 7500 real-time fluorescence quantitative PCR instrument was purchased from Applied Biosystems Inc. (USA).

### Primer design

**Fig 1:**
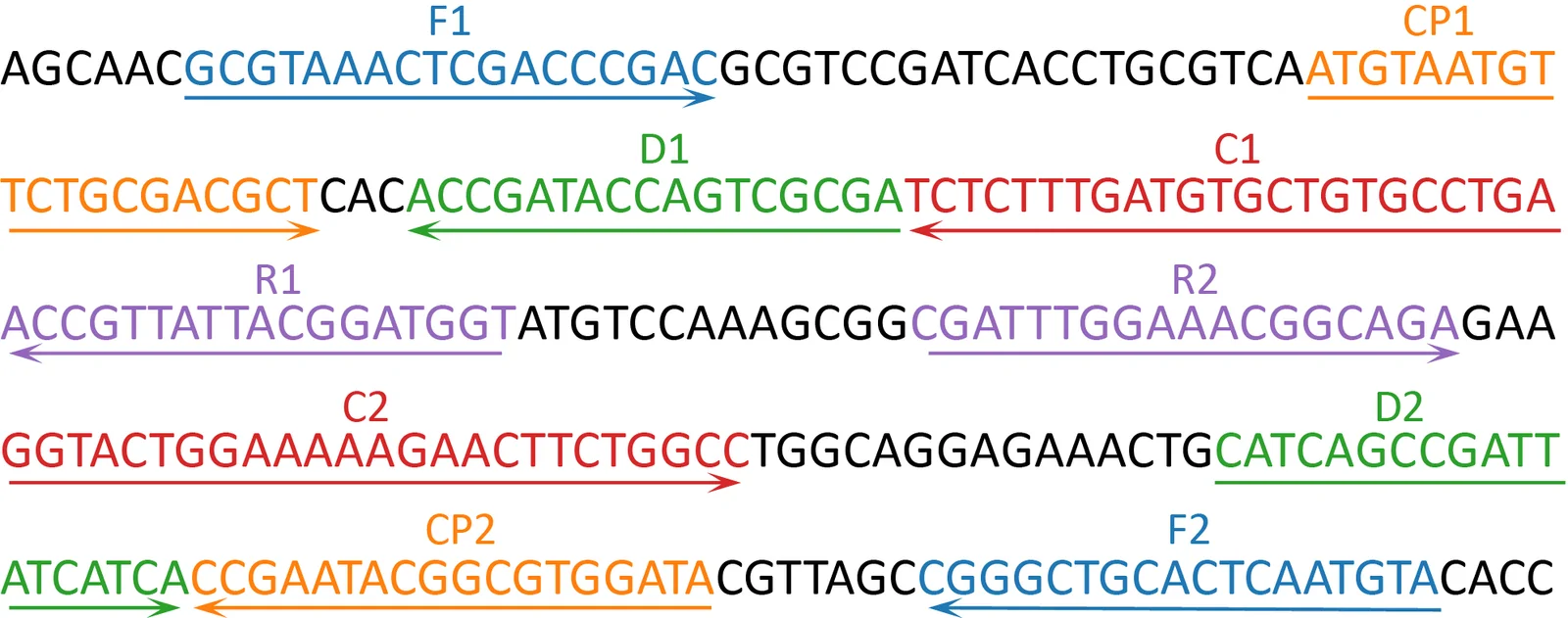
Primer sequences and positions used in this study. Primer positions are indicated by underlines and colored text, and the original primer sequences and their complementary sequences are marked with leftward and rightward arrows, respectively.

Based on the MCDA principle and primer design strategy described by Wang et al. (31). Ten primers were designed using Primer Explorer 5.0 software (https://primerexplorer.jp/lampv5e/index.html), targeting 10 distinct regions within a conserved segment of the *uidA* gene of *Escherichia coli* (GenBank accession number CP009072.1). To identify a suitable target region, *uidA* sequences from multiple *E. coli* genomes were aligned using MEGA, and highly conserved regions were selected as candidate targets. Candidate primers were then analyzed using NCBI Primer-BLAST to minimize potential cross-reactivity with non-target organisms. In addition, primer length, GC content, melting temperature, thermodynamic characteristics, and the potential formation of hairpin structures and primer dimers were evaluated to improve amplification efficiency and stability. The final primer set consisted of two displacement primers (F1, F2), two cross primers (CP1, CP2), and six amplification primers (C1, C2, D1, D2, R1, and R2). In the MCDA-LFB reaction system, primers CP1 and C1 were labeled at their 5′ ends with biotin and fluorescein (FAM), respectively, for LFB visualization. The sequences, positions, and modifications of all primers used in this study are shown in Fig. 1 and Table 1.

**TABLE 1:** Primer sequences and modifications used in this study^*a*^

Primers	Sequence (5′ → 3′)	Length (nt)
F1	GCGTAAACTCGACCCGAC	18
F2	TACATTGAGTGCAGCCCG	18
CP1	TCAGGCACAGCACATCAAAGAGAATGTAATGTTCTGCGACGCT	43
CP1^*b*^	Biotin-TCAGGCACAGCACATCAAAGAGAATGTAATGTTCTGCGACGCT	43
CP2	GGTACTGGAAAAAGAACTTCTGGCCTATCCACGCCGTATTCGG	43
C1	TCAGGCACAGCACATCAAAGAGA	23
C1^*c*^	FAM-TCAGGCACAGCACATCAAAGAGA	23
C2	GGTACTGGAAAAAGAACTTCTGGCC	25
D1	TCGCTGATGGTATCGGT	17
D2	CATCAGCCGATTATCATCA	19
R1	ACCATCCGTAATAACGGT	18
R2	CGATTTGGAAACGGCAGA	18

^*a*^ Biotin: biotin; FAM: 6-carboxyfluorescein; nt: nucleotide.

^*b*^ CP1*: tThis primer is biotinylated at the 5′ end for use in the MCDA-LFB detection system.

^*c*^ C1*: tThis primer is fluorescein-labeled at the 5′ end for use in the MCDA-LFB detection system.

### DNA preparation

Genomic DNA from the reference strain *Escherichia coli* ATCC 25922, clinical isolates, and clinical specimens (whole blood and cerebrospinal fluid [CSF]) was extracted using a commercial nucleic acid extraction and purification kit (Novazene Biotech Co., Ltd.) in accordance with the manufacturer’s instructions. For cultured isolates, bacteria grown overnight on nutrient agar at 37°C were suspended in lysis buffer and subjected to column-based extraction. For clinical specimens, approximately 200 μL of each sample was processed using the same protocol. DNA was eluted in 50 μL of TE buffer and stored at −20°C until use. DNA concentration and purity were measured using a NanoDrop 1000 spectrophotometer. The nucleic acid extraction procedure required approximately 15 min.

### Standard *E. coli*-MCDA-LFB Assay

The standard MCDA reaction was performed in a 25 μL reaction mixture containing: 12.5 μL 2 × reaction buffer, 0.1 μM each of displacement primers F1 and F2, amplification primers C1*, C2, D1, D2, R1, and R2 at 0.2 μM each, cross primers CP1* and CP2 at 0.4 μM each, 1.0 μL Bst 2.0 DNA polymerase (8 U), 1.2 μL VDR, and template DNA (1 μL for DNA from pure culture isolates or 5 μL of purified DNA from clinical specimens), with ultrapure double-distilled water added to a final volume of 25 μL. The reaction mixture was incubated at 63°C for 1 h using a real-time turbidimeter for isothermal amplification. The template added to the reaction included *E. coli* (ATCC 25922) DNA as a positive control, *Streptococcus salivarius* (ATCC 13419) DNA as a negative control, and enzyme-free water as a blank control. Amplification results were assessed using three methods: (i) Real-time turbidity: Reaction curves were monitored using a real-time turbidity meter; turbidity values >0.1 were considered positive, otherwise negative. (ii) Visual dye reaction (VDR): Positive reactions appeared pale green, while negative reactions remained colorless. (iii) Lateral Flow Nanobiosensor Strip (LFB): For LFB detection, 2 μL of amplification product was added to the sample well of the LFB, followed by 80 μL of flow buffer. Results were visually interpreted within 2 min: a red line appearing on both the test line (TL) and control line (CL) indicated a positive result, whereas a red line appearing only on the CL indicated a negative result.

### Optimization of the *E. coli*-MCDA-LFB assay

Genomic DNA from the *E. coli* standard strain was used as the template. Eight MCDA reaction mixtures (25 μL each) were incubated at temperatures ranging from 60°C to 67°C in 1°C increments. Amplification dynamics at each temperature were continuously monitored using a real-time turbidity meter. The reaction speed and amplification efficiency were compared across the different temperatures, and the temperature providing the fastest reaction and highest amplification efficiency was selected as the optimal reaction temperature. At the optimal temperature, amplification times of 10, 20, 30, and 40 min were further evaluated. The LFB method was employed to determine the lowest detectable nucleic acid concentration at each time point, and the shortest reaction time capable of detecting the lowest nucleic acid concentration was chosen as the optimal reaction duration.

### Sensitivity and specificity evaluation of the *E. coli*-MCDA-LFB assay

The initial concentration of genomic DNA extracted from *E. coli* using the nucleic acid extraction kit was measured at 1 ng/μL with a micro-spectrophotometer (NanoDrop 1000). To systematically assess the sensitivity of the assay, DNA from reference strains was serially diluted 10-fold in enzyme-free water, generating a dilution series of 1 ng/μL, 100 pg/μL, 10 pg/μL, 1 pg/μL, 100 fg/μL, 50 fg/μL, and 10 fg/μL. The 50 fg/μL concentration was included as an intermediate gradient to improve detection resolution at low concentrations. A blank control was also included.

One microliter of each DNA dilution was added to separate MCDA reaction mixtures and incubated in a metal bath under the previously determined optimal reaction conditions. The detection sensitivity of the *E. coli*-MCDA-LFB assay was evaluated by determining the limit of detection (LoD) using three methods: real-time turbidimetry, visual dye reaction (VDR), and lateral flow biosensor (LFB). The LoD was defined as the lowest DNA concentration at which the following criteria were met: a turbidity value >0.1 on the real-time turbidimeter, the last appearance of pale green on VDR, and a positive test line on the LFB. Each reaction was performed in triplicate. To evaluate specificity, DNA from 2 *E. coli* strains—including the reference strain ATCC 25922 (nonpathogenic) and a clinical pathogenic strain ST1193 (a lineage associated with neonatal sepsis)—and 28 non*–E*. *coli* strains (Table 2) was used as template DNA. The *E. coli*-MCDA-LFB assay was performed under the same conditions, and all reactions were repeated at least three times.

**TABLE 2:** Strain information used for evaluating the specificity of the *E. coli*-MCDA assay^*a*^

Strain	Source	Number of strains	*E. coli*-MCDA-LFB result
*Escherichia coli*	CDC/CIP	2	P
*Streptococcus agalactiae*	CDC	1	N
*Streptococcus pneumoniae*	CDC	1	N
*Klebsiella pneumoniae*	CDC	1	N
*Listeria monocytogenes*	CDC	1	N
*Haemophilus influenzae*	CDC	1	N
*Staphylococcus aureus*	CDC	1	N
*Staphylococcus epidermidis*	CDC	1	N
*Staphylococcus hominis*	CDC	1	N
*Streptococcus haemolyticus*	CDC	1	N
*Pseudomonas aeruginosa*	CDC	1	N
*Neisseria meningitidis*	CDC	1	N
*Acinetobacter baumannii*	CDC	1	N
*Enterobacter cloacae*	CDC	1	N
*Klebsiella oxytoca*	CDC	1	N
*Klebsiella aerogenes*	CDC	1	N
*Enterococcus faecium*	CDC	1	N
*Citrobacter koseri*	CDC	1	N
*Enterococcus faecalis*	CDC	1	N
*Serratia marcescens*	CDC	1	N
*Acinetobacter pittii*	CDC	1	N
*Acinetobacter johnsonii*	CDC	1	N
*Enterococcus cecorum*	CDC	1	N
*Bordetella pertussis*	CDC	1	N
*Candida albicans*	CDC	1	N
*Mycobacterium tuberculosis*	CDC	1	N
*Epstein-Barr virus*	CDC	1	N
*Respiratory syncytial virus*	CDC	1	N
*Influenza A virus*	CDC	1	N

^*a*^ CDC, Chinese Center for Disease Control and Prevention; CIP, Capital Institute of Pediatrics; N, negative; P, positive.

### Clinical application assessment of the *E. coli*-MCDA-LFB assay

To validate the clinical feasibility of this method, we collected 75 sterile body fluid specimens—including 58 blood and 17 cerebrospinal fluid (CSF) samples—from neonates who were clinically diagnosed with sepsis or bacterial meningitis and in whom *E. coli* infection was considered highly likely on the basis of the overall clinical assessment, including clinical manifestations, routine laboratory findings, perinatal or in-hospital risk factors, and the treating clinicians’ initial judgment. All patients were admitted to the Neonatal Department of Capital Institute of Pediatrics between November 2024 and November 2025. The neonates had a median age of 6 days, including 42 males and 33 females. Blood specimens were drawn into EDTA anticoagulant tubes, and all samples were either processed within 4 h of collection or stored at −80°C until nucleic acid extraction, with no specimen undergoing more than one freeze-thaw cycle. This was therefore a clinically enriched cohort rather than an unselected consecutive series of all neonatal sepsis cases. However, enrollment was not based on prior definitive microbiological confirmation. These specimens were analyzed using conventional culture, qPCR, and the MCDA-LFB assay, and the results were subsequently compared. For the MCDA-LFB assay, each sample was tested in three technical replicates, and the assay was repeated on independently extracted DNA from the same specimen to account for biological variation.

### Statistical analysis

Statistical analyses were conducted using SPSS 26.0, with a significance level of *α* = 0.05. Using “culture-positive or qPCR-positive” as the composite reference standard, the sensitivity, specificity, positive predictive value (PPV), negative predictive value (NPV), and 95% confidence intervals (CIs, calculated using the Clopper-Pearson method) were determined for each detection method. Inter-method agreement was evaluated using the Kappa coefficient.

## RESULTS

### Principle of the *E. coli*-MCDA-LFB assay

**Fig 2:**
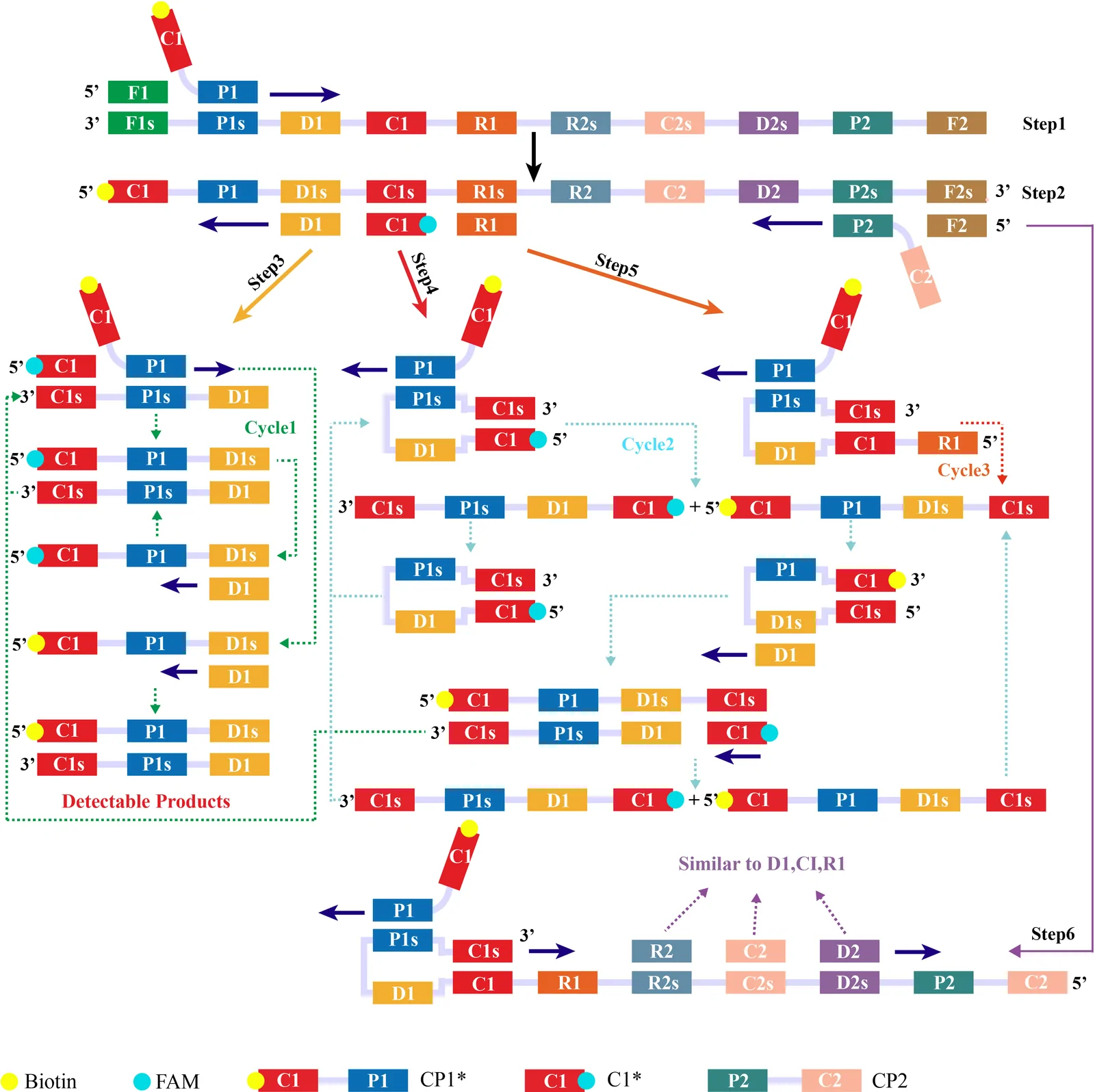
Mechanistic description of the *E. coli*-MCDA amplification. Step 1: The cross primer CP1 (C1 + P1) anneals to the P1s region of the target sequence and initiates strand synthesis. The newly synthesized strand is subsequently displaced by extension from the upstream displacement primer F1, generating a single-stranded intermediate template. Step 2: Amplification primers (D1, C1, and R1), together with cross primer CP2 (C2 + P2) and displacement primer F2, hybridize to their corresponding target regions on the newly formed strand. Bst DNA polymerase extends these primers simultaneously, producing multiple intermediate amplicons through coordinated strand displacement reactions. The extension products containing D1, C1, and R1 sequences serve as new templates for further primer annealing. Successive strand displacement events result in the formation of structured intermediates capable of self-primed cycling amplification. Step 3: The D1-derived product is displaced by C1-initiated synthesis, entering the first cyclic amplification process (Cycle 1). This generates additional target-specific amplicons containing primer-labeled regions. Steps 4 and 5: Similar to the D1-derived structure, the C1- and R1-derived products initiate independent cyclic amplification events (Cycle 2 and Cycle 3). These coordinated cycles continuously amplify the target sequence through repeated strand displacement and re-priming. Step 6 and subsequent steps: The newly generated products act as templates for further rounds of elongation and strand displacement, resulting in exponential amplification of target fragments. Selected primers are labeled with FAM or biotin, producing dual-labeled amplicons that are captured by the LFB for visual detection on the test strip.

**Fig 3:**
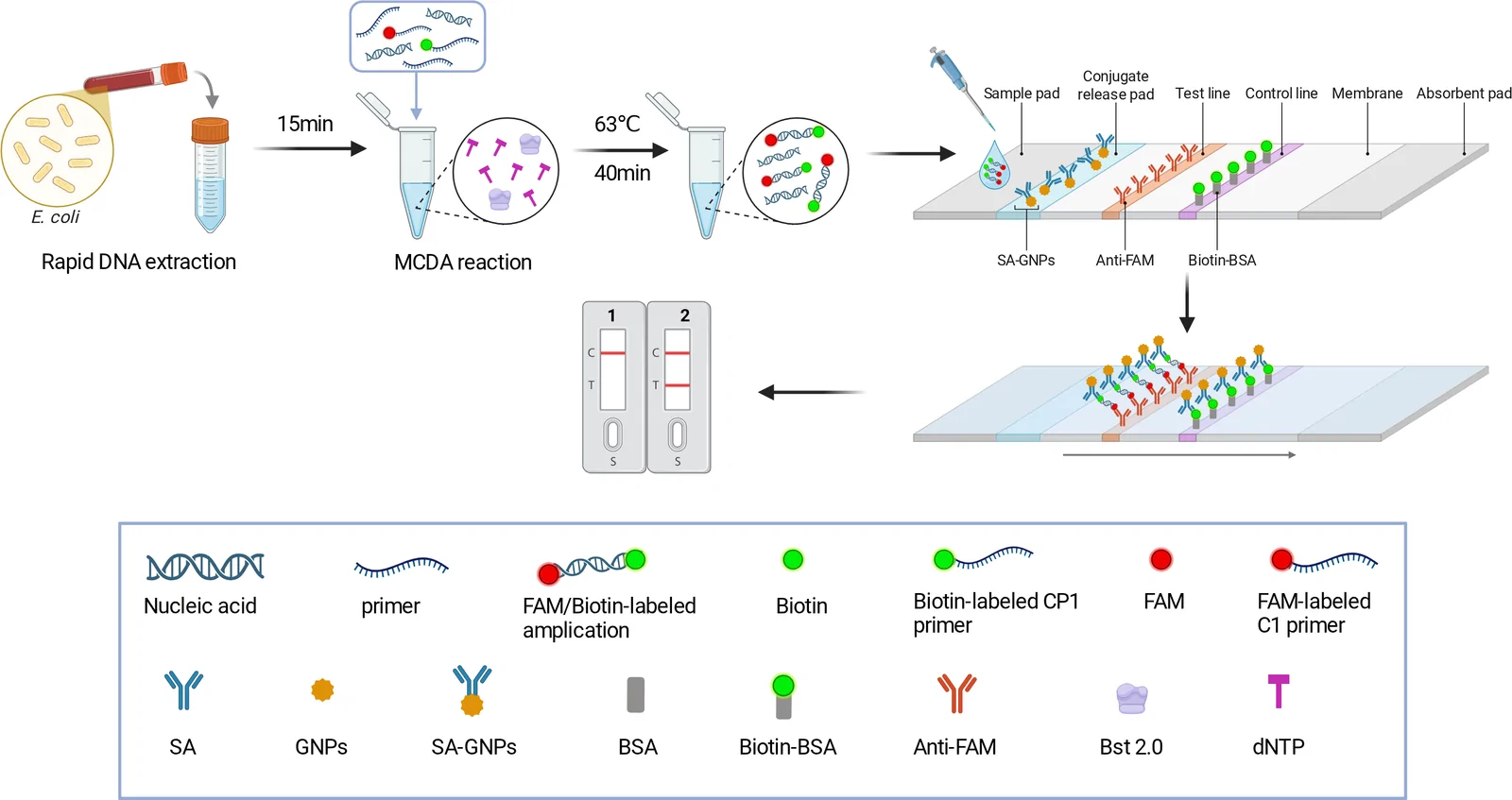
Schematic illustration of the *E. coli*-MCDA-LFB assay. FAM-labeled C1 and biotin-labeled CP1 primers were used to generate double-labeled amplicons. Amplicons were captured by immobilized anti-FAM on the LFB and visualized through interaction with streptavidin-coated red nanoparticles (SA-GNPs), producing a red line at the TL region. Excess SA-GNPs were captured by immobilized biotin-BSA at the CL region, producing a red control line.

In this study, we developed an *E. coli*-MCDA-LFB assay combining MCDA with an LFB for rapid, visual detection of *E. coli*. Unlike conventional molecular assays, this method allows direct observation of results without the need for sophisticated laboratory instrumentation. As illustrated in Fig. 2 and 3, the reaction utilizes specific primers targeting the *uidA* gene, including a FAM-labeled C1 primer (C1*) and a biotin-labeled CP1 primer (CP1*). Isothermal amplification at 63°C for 40 min generates abundant amplicons incorporating both FAM and biotin, which serve as the basis for subsequent LFB detection.

The LFB strip consists of a sample pad, binding pad, nitrocellulose membrane (reaction zone), and absorbent pad (Fig. 3). When amplification products and buffer are added to the sample pad, the liquid migrates via capillary action. In the binding pad, streptavidin-coated gold nanoparticles (SA-GNPs) specifically bind biotin moieties on the amplicons, forming complexes that continue migrating. Upon reaching the test line (TL), anti-FAM antibodies capture the FAM-labeled products, causing SA-GNP accumulation and a visible red line. Unbound SA-GNPs are subsequently captured by biotinylated bovine serum albumin (biotin-BSA) on the control line (CL), forming a second red line. Thus, the presence of *E. coli* DNA is indicated by red lines at both the TL and CL, whereas target absence results in a red line only at the CL, enabling rapid, instrument-free interpretation.

### Effectiveness of the primer set for the *E. coli*-MCDA-LFB assay

**Fig 4:**
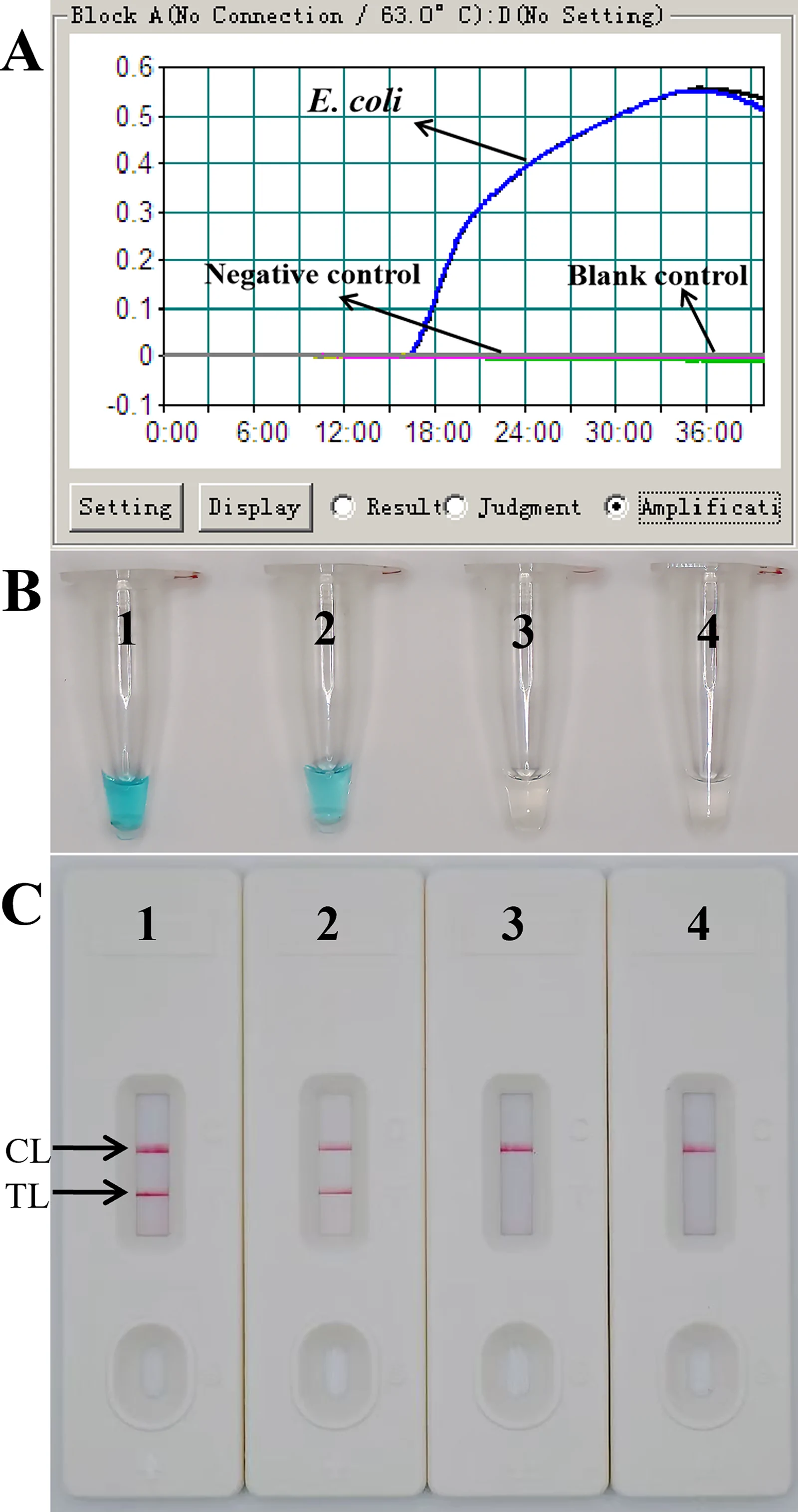
Validation of the *E. coli*-MCDA-LFB assay. Analysis of amplified products by real-time turbidity measurement (**A**). The vertical axis indicates turbidity readings (FTU), and the horizontal axis shows the reaction time (minutes). VDR (**B**) and LFB (**C**) results for the MCDA assay. CL, control line; TL, test line. Lanes 1 and 2: *E. coli* nucleic acid. Genomic DNA for both *E. coli* samples (Lanes 1 and 2) was standardized to 1 ng per reaction. Lane 3: *Streptococcus salivarius* (negative control). Lane 4: blank control (double-distilled water, DW).

To validate the primers, amplification products were analyzed using the three distinct detection methods. Real-time turbidity confirmed that reactions containing *E. coli* DNA exceeded the 0.1 threshold (positive), whereas *Streptococcus salivarius* and nuclease-free water controls remained below 0.1 (negative; Fig. 4A). Similarly, VDR produced a pale green color only in the positive control, with negative and blank controls remaining colorless (Fig. 4B). LFB detection displayed red lines at both the TL and CL for the positive control, but only at the CL for negative samples (Fig. 4C). These results confirm the suitability of the MCDA primers and the assay for the detection of *E. coli*.

### Optimal reaction conditions for the *E. coli*-MCDA-LFB assay

**Fig 5:**
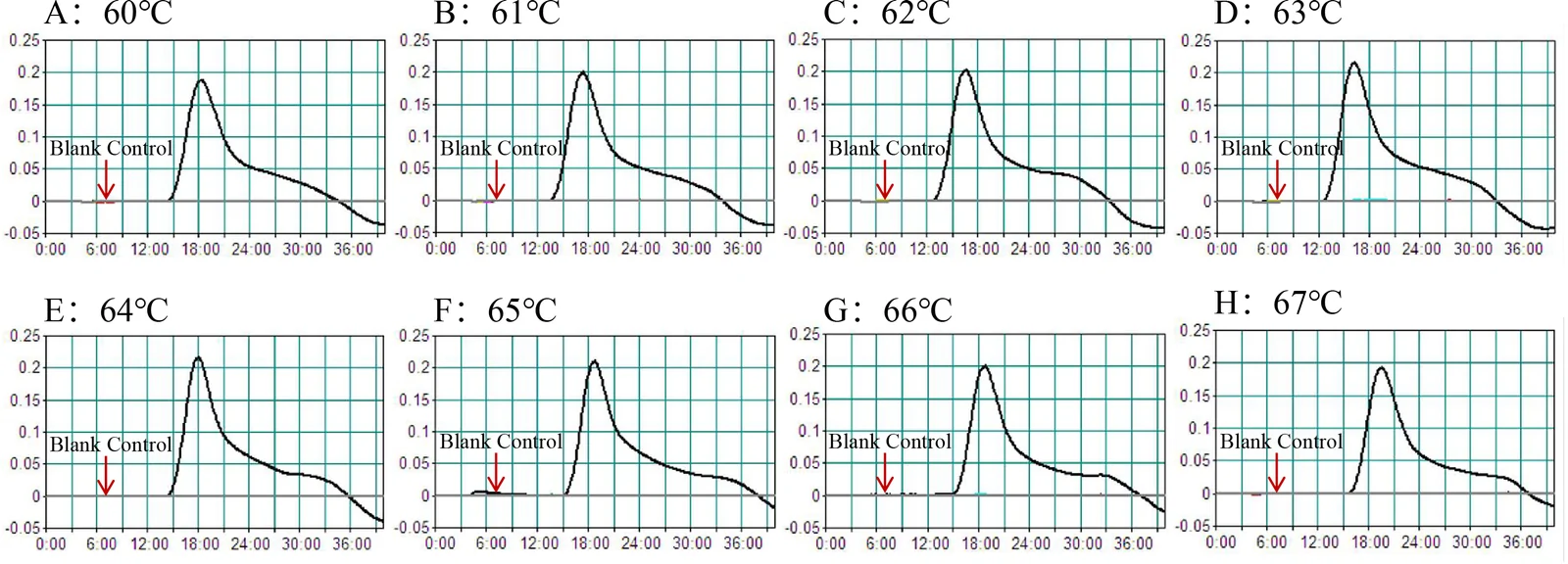
Amplification efficiency of the *E. coli*-MCDA assay at different temperatures. Real-time turbidity monitoring (threshold set at 0.1; values > 0.1 considered positive) generated eight kinetic curves (**A–H**) under a temperature gradient from 60°C to 67°C, with data recorded at 1°C intervals. The concentration of target *E. coli* DNA was maintained at 50 fg/reaction throughout the experiment.

Real-time turbidity analysis was performed to evaluate amplification performance across a temperature gradient (Fig. 5). Positive amplification was observed at multiple temperatures, and the overall amplification profiles were comparable. Among the tested conditions, 63°C (Group D) showed an earlier increase in turbidity and a relatively high peak signal compared with the other temperatures. Although the differences were modest, 63°C demonstrated slightly faster amplification under our experimental conditions and was therefore selected for subsequent assays.

**Fig 6:**
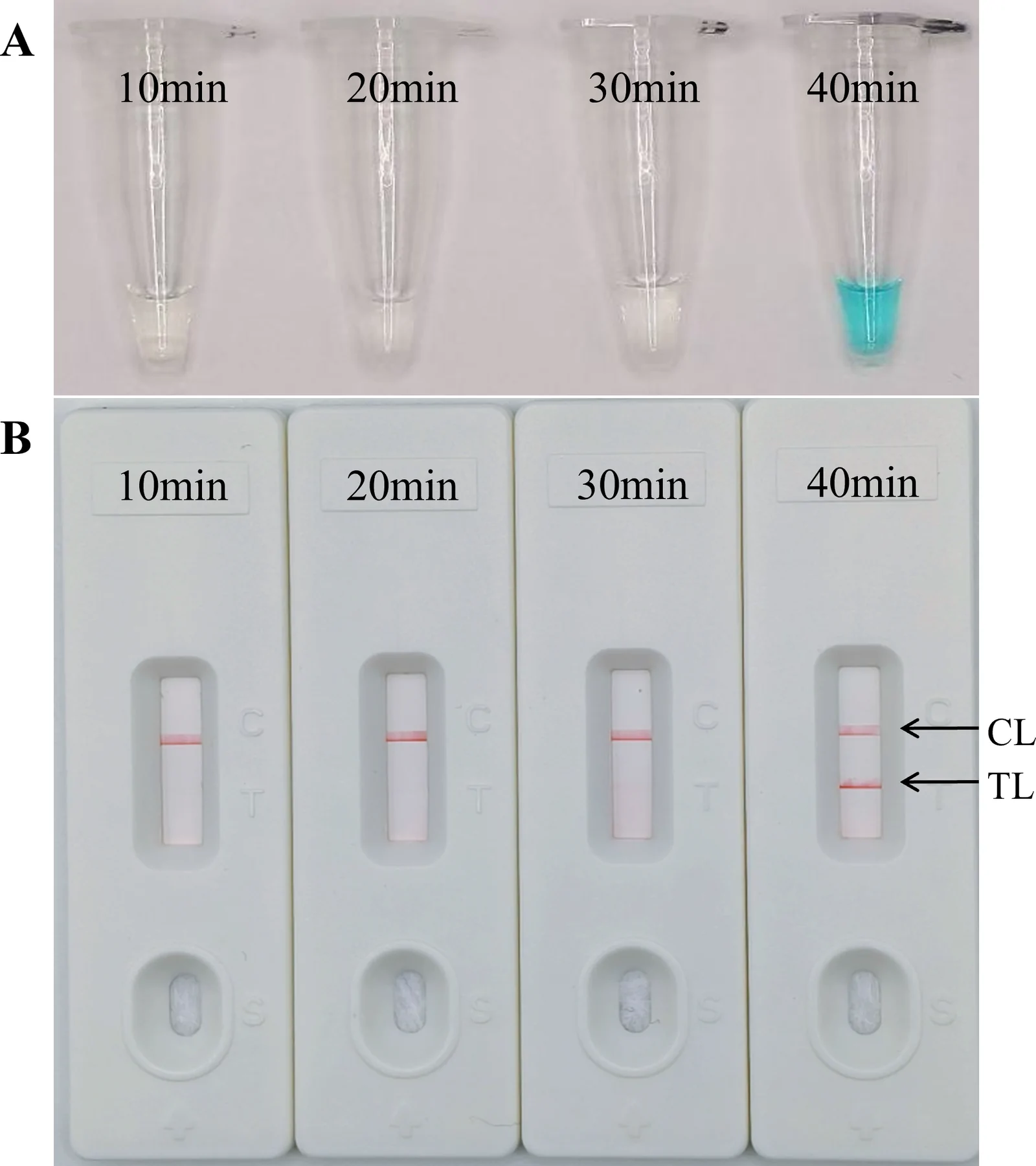
Detection capability of the *E. coli*-MCDA assay at different amplification times. At the optimal amplification temperature (63°C), four different reaction times (10, 20, 30, and 40 min) were evaluated using 50 fg of *E. coli* DNA. Results were monitored using VDR (**A**) and LFB (**B**). CL, control line; TL, test line.

To determine the optimal reaction time, *E. coli* DNA at the LoD level (50 fg/reaction) was evaluated at 63°C. Stable positive signals were detected within 40 min (Fig. 6). Accordingly, 40 min was defined as the optimal reaction time for the *E. coli*-MCDA-LFB assay.

### Sensitivity of the *E. coli*-MCDA-LFB assay

**Fig 7:**
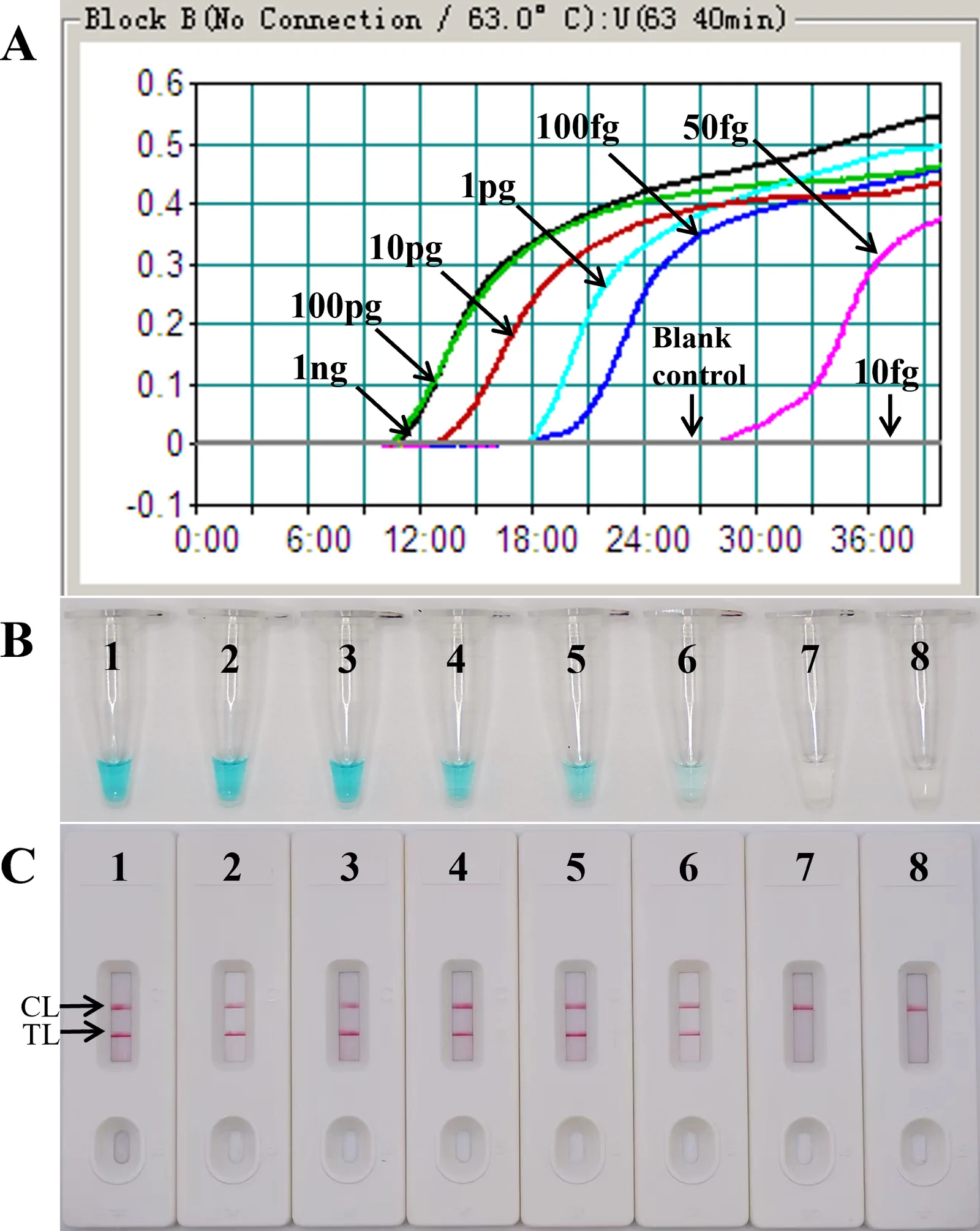
Sensitivity of the *E. coli*-MCDA-LFB assay. Serial dilutions of the target DNA (1 ng/μL, 100 pg/μL, 10 pg/μL, 1 pg/μL, 100 fg/μL, 50 fg/μL, and 10 fg/μL) were subjected to MCDA reactions. Results were monitored using three detection modes: real-time turbidity (**A**), VDR (**B**), and LFB (**C**). Tubes B1–B7 and LFB strips C1–C7 correspond to detection outcomes for *E. coli* DNA ranging from 1 ng/μL to 10 fg/μL. Tube B8 and strip C8 served as blank controls. CL, control line; TL, test line.

Sensitivity was assessed across eight reaction systems (Fig. 7). LFB analysis displayed two red lines (TL and CL) for template amounts ranging from 1 ng to 50 fg, indicating positive results, whereas 10 fg and the blank control showed only the CL. Visual inspection confirmed that while the reaction color lightened as concentrations decreased from 1 ng to 50 fg, a faint light-green hue remained visible. Conversely, the 10 fg sample and blank control appeared colorless, indistinguishable from negative controls. Thus, the LoD for the *E. coli*-MCDA-LFB system was determined to be 50 fg/reaction, which compares favorably to the ~450 fg limit of conventional PCR (36).

### Specificity of the *E. coli*-MCDA-LFB assay

**Fig 8:**
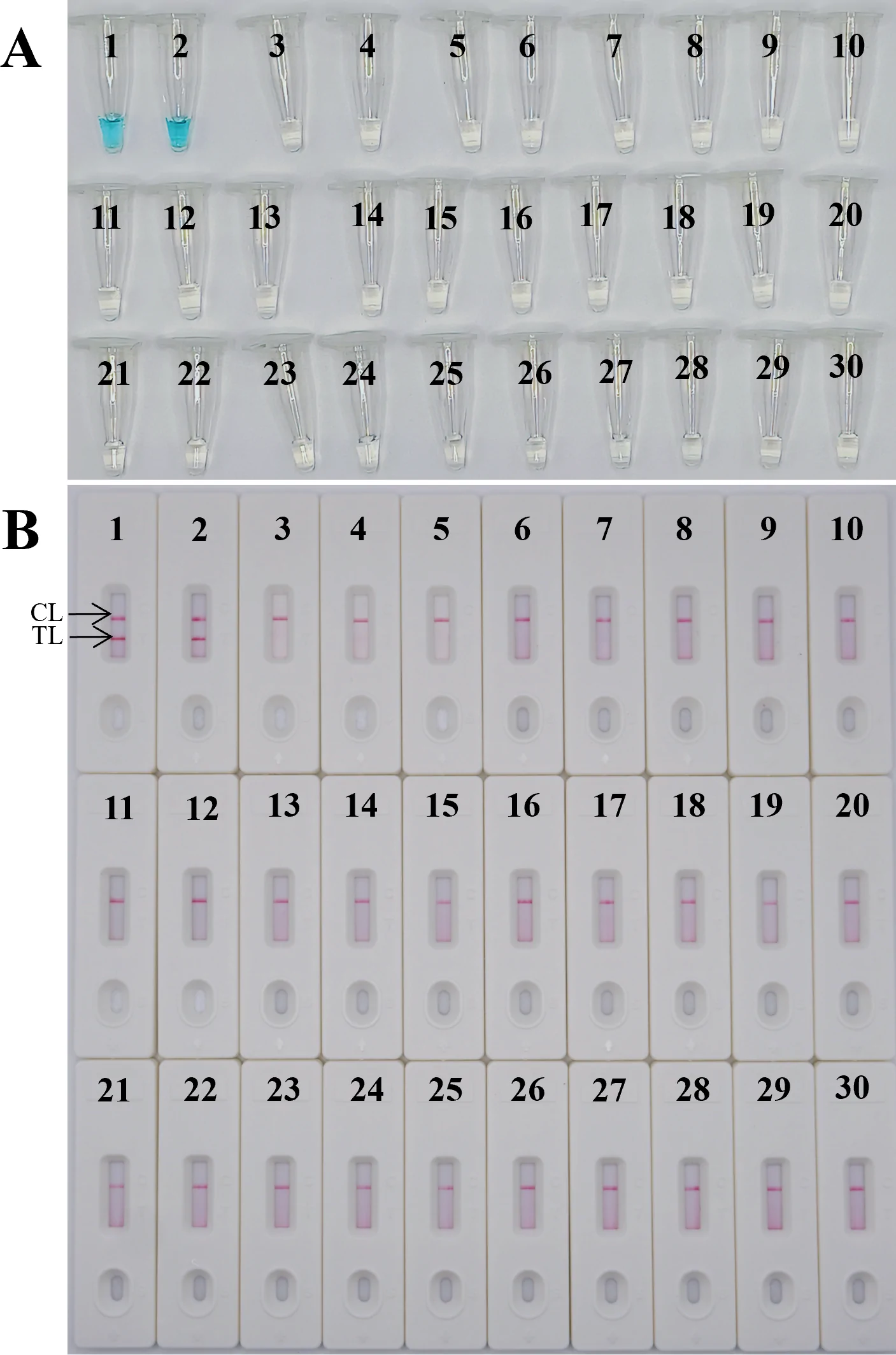
Specificity of the *E. coli*-MCDA-LFB assay. Detection specificity was evaluated using 28 non*–E*. *coli* pathogens. Genomic DNA from all samples was standardized to 1 ng per reaction. Amplification products were analyzed by VDR (**A**) and LFB (**B**). Test tubes/LFB samples 1 and 2 correspond to *E. coli* and showed positive results, whereas samples 3–30, representing 28 non*–E*. *coli* strains, were all negative. CL, control line; TL, test line.

Analytical specificity was evaluated using 30 microbial species, comprising 2 *E. coli* and 28 non*–E*. *coli* strains. As depicted in Fig. 8, both *E. coli* strains produced positive results, evidenced by pale green reaction tubes and positive LFB bands. In contrast, all 28 non*–E*. *coli* strains yielded negative results. These data indicate that the *E. coli*-MCDA-LFB assay showed 100% analytical specificity against the tested non–*E*. *coli* panel, with no cross-reactivity observed under the conditions evaluated.

### Application of the *E. coli*-MCDA-LFB assay in clinical specimens

**Fig 9:**
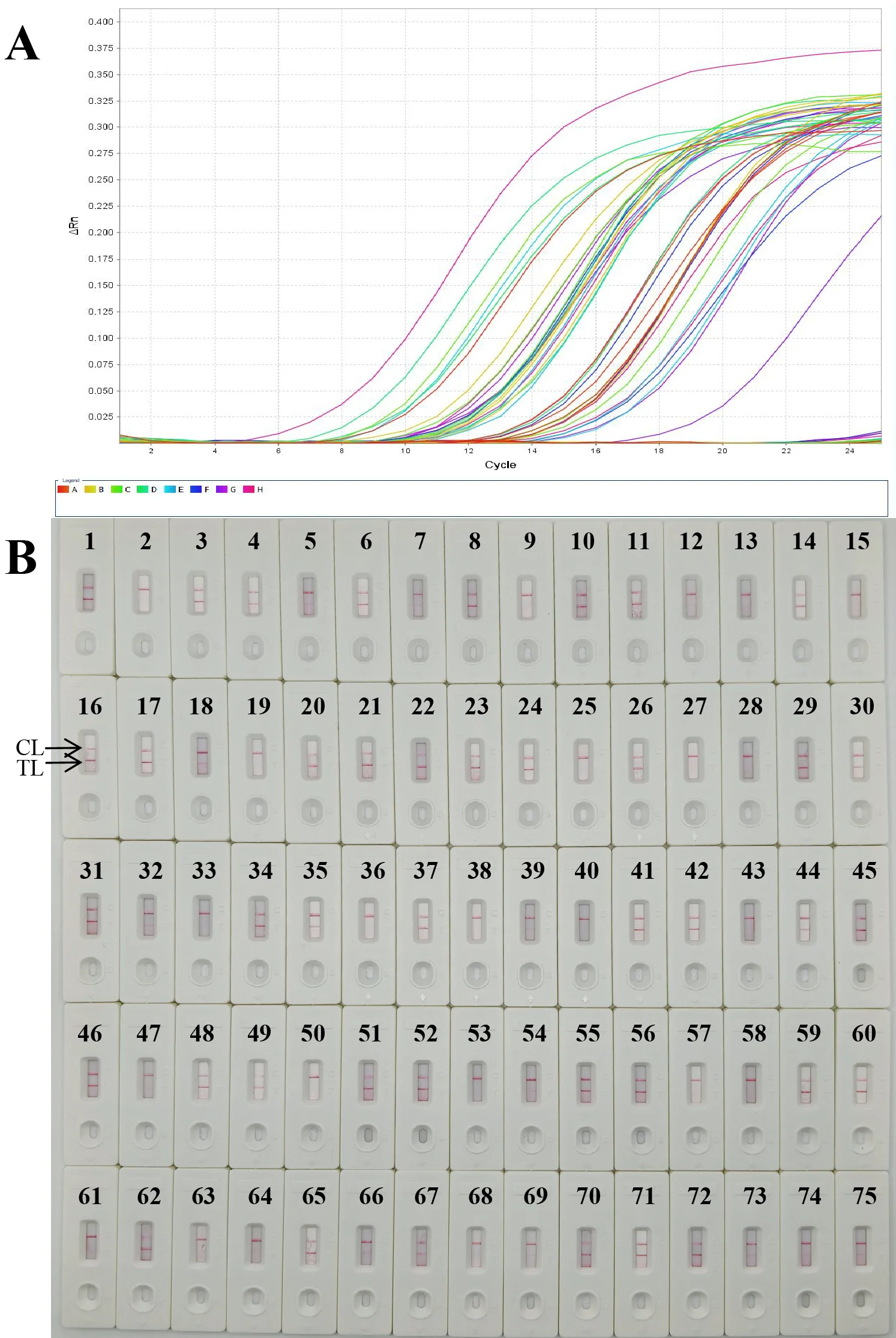
Clinical validation of the *E. coli*-MCDA-LFB assay. Amplified products were analyzed by qPCR (**A**) and LFB (**B**). Tubes 1–75 in the LFB assay represent clinical samples with suspected *E. coli* infection. CL, control line; TL, test line.

Seventy-five clinical specimens were analyzed using conventional culture, qPCR, and the MCDA-LFB method. As shown in Fig. 9, positive detection rates were 14.7% (11/75) for culture, and 56.0% (42/75) for both qPCR and MCDA-LFB. The results of MCDA-LFB were fully concordant with qPCR (*κ* = 1.000). When conventional culture was used as the reference standard, MCDA-LFB demonstrated a sensitivity of 100.0% (11/11; 95% CI: 71.5–100.0%) and a specificity of 51.6% (33/64; 95% CI: 38.7–64.2%). The positive predictive value and negative predictive value were 26.2% (11/42; 95% CI: 14.6–41.1%) and 100.0% (33/33; 95% CI: 89.4–100.0%), respectively (Table 3). Using a composite reference standard (culture-positive or qPCR-positive), the MCDA-LFB assay achieved 100.0% sensitivity (95% CI: 91.6–100.0%), 100.0% specificity (95% CI: 89.4–100.0%), 100.0% PPV, and 100.0% NPV. Chi-square analysis confirmed that MCDA-LFB detected significantly more positives than culture (χ² = 31.6, *P* < 0.001), highlighting the substantially higher detection rate of molecular methods compared with conventional culture.

**TABLE 3:** Diagnostic performance of MCDA-LFB using conventional culture as the reference standard

MCDA-LFB	Culture positive	Culture negative	Total
Positive	11	31	42
Negative	0	33	33
Total	11	64	75

## DISCUSSION

Invasive neonatal infections constitute a primary cause of global morbidity and mortality (1), responsible for approximately 36% of the 4 million annual neonatal deaths (37). *E. coli* stands as a principal agent of invasive bacterial disease and the most frequent gram-negative pathogen isolated in neonatal sepsis (38). Epidemiological data indicate that *E. coli* is one of the dominant causes of neonatal bacteremia, accounting for 34.01% of cases (39). Risks are notably elevated in preterm and extremely low-birth-weight infants, where incidence exceeds 10 per 1,000 live births and case-fatality rates approach 40%.^40^ Despite this burden, diagnosis relies heavily on conventional blood culture, a protracted method lacking the immediacy required for acute clinical management. As a result, empirical antibiotic administration remains the standard of care, leading to extended broad-spectrum exposure. This practice accelerates antimicrobial resistance and risks adverse effects (11), evidenced by rising resistance rates in *E. coli* to first-line regimens like ampicillin and aminoglycosides (39, 41, 42). Therefore, the development of rapid, sensitive diagnostic tools for *E. coli* is critical for enabling early and appropriate intervention.

While blood culture remains the diagnostic standard, it requires 24–72 h and offers reduced sensitivity in contexts of prior antibiotic use or low-level bacteremia (11, 13). To achieve optimal detection in neonates, sufficient blood volumes are necessary to capture low bacterial loads (1–10 CFU/mL) (13). Although molecular methods like qPCR provide high specificity, their reliance on thermal cycling infrastructure limits their utility for point-of-care testing (POCT) (17). Metagenomic next-generation sequencing (mNGS) offers comprehensive detection but is constrained by high costs, complex workflows, and contamination susceptibility (20). Furthermore, while various isothermal amplification techniques exist, such as LAMP and NASBA, they often fail to fully satisfy WHO criteria for POCT (43, 44). Conversely, MCDA overcomes these limitations by facilitating rapid, specific nucleic acid amplification at constant temperatures (61–69°C). This process eliminates the need for thermal cyclers, completing reactions within 40 min using basic heating devices (31). Consequently, MCDA has gained traction in clinical and food safety applications (45–47).

Mechanistically, MCDA utilizes distinct primer sets targeting multiple genomic regions to drive strand-displacement amplification under isothermal conditions (31). This design ensures the rapid generation of abundant amplicons while maintaining exceptional specificity. Building on this, variants like endonuclease restriction–mediated MCDA have been developed to support multiplexing (43). More recently, the integration of MCDA with nanoparticle-based LFB has enabled instrument-free visual interpretation. Due to their unique optical properties and surface effects, nanoparticles facilitate the direct detection of labeled amplicons within two minutes.^48^ This streamlined workflow removes the need for complex equipment, presenting a cost-effective solution for POCT (49). For instance, the detection of Streptococcus agalactiae via MCDA-LFB can be completed in approximately 50 minutes (26). Such protocols simplify testing procedures, aligning with requirements for rapid on-site diagnosis (50). Indeed, MCDA-LFB has demonstrated accuracy in detecting diverse pathogens, including *Haemophilus influenzae* and *Candida albicans* (21, 23, 25–27).

To date, the application of MCDA-LFB for *E. coli* diagnosis in neonatal sepsis has not been described. Here, we established an *E. coli*-MCDA assay targeting the species-specific *uidA* gene, optimizing conditions to 63°C for 40 min. Integrating MCDA with LFB, the complete workflow (from extraction to readout) is accomplished within a clinically relevant timeframe. Our process requires approximately 50–60 min total, offering a substantial speed advantage over the ~2 h needed for conventional PCR. Thus, the MCDA-LFB platform streamlines operations and minimizes complexity. Significantly, our 50 fg/reaction limit of detection (LoD) is markedly lower than that of conventional PCR (~450 fg). This heightened sensitivity helps prevent missed diagnoses and facilitates appropriate antibiotic stewardship. Furthermore, our analytical sensitivity parallels or exceeds reported values for other pathogens, such as 300 fg for *S. agalactiae* (26) and 100 fg for *H. influenzae* (51). The analytical sensitivity of the assay was 50 fg per reaction, corresponding to approximately 10 genome equivalents. Importantly, the template added was purified DNA extracted from clinical samples, not direct whole blood or CSF. Therefore, this value does not reflect actual bacterial concentrations in neonatal blood or CSF, which are generally below 50 CFU/mL (52). DNA extraction from larger sample volumes concentrates target nucleic acids prior to amplification, increasing the effective template concentration in the reaction (53). Nevertheless, detection of extremely low-level bacteremia may remain challenging, and further optimization of sample preparation or template input could improve clinical sensitivity.

Analytical specificity testing showed reactivity exclusively with *E. coli* among the species evaluated. When conventional culture was used as the reference standard, MCDA-LFB exhibited high sensitivity but reduced specificity, primarily due to additional cases detected by molecular methods that were culture-negative. In neonatal sepsis, culture sensitivity is frequently compromised by prior antibiotic exposure, small blood volumes, and low bacterial burden. The relatively high molecular positivity rate (56.0%) observed in this study may therefore reflect the limited sensitivity of culture rather than overestimation by molecular assays. Clinically, MCDA-LFB showed complete concordance with qPCR (*κ* = 1.0) and detected more positive cases than conventional culture. Consistent with previous reports in neonatal central nervous system infections, PCR-based methods have been shown to achieve higher pathogen detection rates than culture, particularly in specimens obtained after antibiotic administration, as they are less dependent on bacterial viability (54). Nevertheless, molecular-positive/culture-negative findings should be interpreted with caution, as they may represent true infection, detection of non-viable organisms following antibiotic exposure, or, less commonly, contamination despite strict laboratory controls. The *uidA* gene is a conserved species-level marker and does not distinguish pathogenic from commensal *E. coli* strains. Although blood and cerebrospinal fluid are normally sterile, and detection of *E. coli* DNA in these specimens is generally clinically meaningful, incorporation of virulence-associated targets could further improve diagnostic precision.

Several limitations merit consideration. The single-center design and limited sample size (*n* = 75) require validation in larger, multicenter studies. In addition, the analytical limit of detection was determined using serial dilutions of purified genomic DNA rather than clinical samples spiked with known bacterial concentrations. Matrix-associated inhibitors present in whole blood or cerebrospinal fluid may affect amplification efficiency (55). Therefore, the analytical sensitivity observed under clean conditions may differ from that in complex clinical specimens. Given the diverse etiology of neonatal sepsis, multiplex assay development would enhance diagnostic coverage. While the amplification step requires only a simple heating device, nucleic acid extraction currently relies on centrifugation-based purification, which may limit fully equipment-independent point-of-care deployment. This represents a practical limitation of the present study. In future applications, simplified lysis-based extraction methods or integrated sample-to-answer platforms may further improve feasibility in resource-limited settings.

### Conclusion

We established a visual MCDA-LFB assay for the rapid, sensitive detection of *E. coli*. The method achieved a 50 fg detection limit and high analytical specificity against the tested non–*E*. *coli* organisms. The entire workflow is completed within one hour without complex instrumentation. Compared with conventional culture, this assay demonstrated a higher detection rate and a substantially shorter turnaround time, while showing complete agreement with qPCR in this cohort. These attributes highlight its potential for improving early diagnosis and clinical decision-making, particularly in resource-limited settings.

## Data Availability

All data generated and analyzed during this study are included in this published article and its additional information.
